# Which variables are associated with blood glucose levels outside the target range in surgical critically ill patients? A retrospective observational study

**DOI:** 10.1186/1754-9493-5-5

**Published:** 2011-04-06

**Authors:** Manfred Weiss, Martina Kron, Birgit Hay, Michael Taenzer, Peter Radermacher, Michael Georgieff

**Affiliations:** 1 Department of Anaesthesiology, University Hospital Medical School Ulm, Steinhoevelstr. 9, 89075 Ulm, Germany; 2 Institute of Epidemiology and Medical Biometry, University Ulm, Schwabstr. 13, 89075 Ulm, Germany

## Abstract

**Background:**

The aim of the present study is to determine the variables affecting blood glucose concentrations outside the target range of 80 and 150 mg/dl in critically ill surgical patients.

**Methods:**

All critically ill surgical patients admitted to a university ICU, from 01/2007 to 12/2008, were surveyed daily using computer assistance with respect to minimal and maximal daily blood glucose concentrations, application of insulin and demographic/clinical variables. Multiple logistic regression for clustered data with backward elimination was performed to identify variables strongly associated with blood glucose concentrations < 80 mg/dl or ≥ 150 mg/dl in 804 patients with an ICU stay > 72 hours.

**Results:**

Application of insulin (odds ratio (OR) 2.1, with corresponding 95% confidence interval (CI) 1.7; 2.6), noradrenaline (OR 1.4, 95% CI 1.2 - 1.8) or steroids (1.3, 1.003 - 1.7), and age (per year) (1.02, 1.01 - 1.03) were associated with an increased risk of blood glucose concentrations < 80 mg/dl. In analogy, application of insulin (OR 2.4, 95% CI 2.0 - 2.7), noradrenaline (1.4, 1.2 - 1.6) or steroids (1.4, 1.2 - 1.7), severe sepsis (1.2, 1.1 - 1.4), neurosurgery (OR 1.0) compared to abdominal, vascular and trauma surgery, and age (per year) (1.01, 1.01 - 1.02), were associated with an increased risk of blood glucose concentrations ≥ 150 mg/dl.

**Conclusions:**

Critically ill surgical patients are at an increased risk for fluctuating blood glucose concentrations ranging < 80 mg/dl or ≥ 150 mg/dl in particular if they are of advanced age and require administration of insulin, noradrenaline, and/or steroids. Patients who underwent neurosurgery and/or presented with severe sepsis/shock are those in particular at risk for blood glucose concentrations ≥ 150 mg/dl.

## Background

In 2004 and 2008, the "Surviving Sepsis Campaign" (SSC) guidelines for the management of severe sepsis and septic shock were published to improve outcome of critically ill patients [1,2]. These guidelines recommend that patients with severe sepsis/septic shock and blood glucose concentrations ≥ 150 mg/dl receive IV insulin therapy to keep blood glucose levels in the range of 80 - 150 mg/dl [1,2]. A validated protocol for insulin dose adjustments was suggested targeting glucose levels of < 150 mg/dl [1,2].

Prophylactic perioperative intensive insulin therapy (IIT) reduced mortality and organ dysfunctions [3] in surgical patients, with a length of stay in the intensive care unit (ICU) of at least three days [4], but not in a combined population of medical/surgical patients [5], nor in patients with severe sepsis [6]. In contrast to the first two successful trials [3,4], five randomized controlled trials showed no beneficial effects of strict glycemic control with intensive insulin therapy [5-8]. Some reasons for these contrasting observations are due to variability in the performance of strict glycemic control, differences among trial designs, changes in standard of care, time initiation of strict glycemic control, and the convergence between the intervention groups and control groups with respect to achieved blood glucose levels in the successive randomized controlled trials [7]. Large variability of blood glucose concentrations in IIT and control groups was considered as one possible explanation for the lack of beneficial effects of insulin therapy [9,10]. Moreover, patients with sepsis and increased glycemic lability index, but lower average glucose values, had an almost five-fold increased risk of hospital mortality (odds ratio = 4.73, 95% confidence interval = 2.6 - 8.7) [11]. It remained unclear whether the increased susceptibility to severe hypoglycemia (glucose level ≤ 40 mg/dl) with intensive insulin therapy in sepsis patients was due to liver and renal dysfunction [6]. Taken together, recognizing predisposing factors for blood glucose concentrations < 80 mg/dl or ≥ 150 mg/dl might help in identifying patients at risk so that more intensive monitoring or less tight blood glucose targets could be used.

In this context, the following variables have been reported to be associated with increased risk for hypoglycemia or hyperglycemia: age [12,13], sex [12], type of surgery; severity of disease [14] and severe systemic inflammatory response syndrome (SIRS)/sepsis [15,16]; kidney [14,17], liver [17] and organ dysfunctions; application of steroids [13,18-21], adrenaline and/or noradrenaline infusion [16,21,22]. Nonetheless, it was unclear which of these factors are most responsible in hindering the efforts to maintain blood glucose levels in the range of 80 - 150 mg/dl as adviced by the SSC guidelines [1,2]. Therefore, the present study was performed in a surgical adult intensive care unit (ICU) in surgical critically ill patients with an ICU stay > 72 hours to answer the following questions:

1. What is the frequency of observed blood glucose levels outside the target range of 80 - 150 mg/dl?

2. Is age, gender, type of surgery, severity of disease, severity of organ dysfunctions, application of steroids, adrenaline, noradrenaline, steroids or insulin, and severe sepsis and shock associated with blood glucose concentrations < 80 mg/dl or ≥ 150 mg/dl, respectively?

3. What factors are most responsible for blood glucose levels outside the target range?

## Methods

### Patients and data collection

The prospective study is in compliance with the Helsinki declaration and was approved by the Independent Ethics Committee of the University Ulm, which waived informed consent because this was an observational study, and no additional interventions were performed.

All surgical critically ill patients, from 01/2007 to 12/2008, admitted to the Anesthesiology ICU of the University Hospital Ulm after major trauma, vascular, lung, brain and abdominal surgery were followed daily, computer assisted, for minimal and maximal blood glucose concentrations.

The following variables were documented on admission: age, sex, type of surgery (major trauma, vascular and lung, brain or abdominal surgery), and severity of disease by the Simplified Acute Physiology Score II (SAPS II) score [23]. The severity of the systemic inflammatory response syndrome (SIRS)/sepsis and shock (the 1992 American College of Chest Physicians/Society of Critical Care Medicine (ACCP/SCCM) consensus conference definitions [24] and the 2003 SCCM/ESICM/ACCP/ATS/SIS sepsis definitions [15]), acute kidney injury demanding extracorporeal renal support, liver dysfunction assessed by the Model of Endstage Liver Disease (MELD) score [25], organ dysfunctions reflected by the Sequential Organ Failure Assessement (SOFA) score [26], insulin administration, steroid, adrenaline and/or noradrenaline infusion, were monitored, computer-assisted, on a daily basis by the ICU residents and staff physicians. SIRS was defined by two or more of the four conditions: temperature, heart rate, respiratory rate, and white blood cell count (WBC) by the 1992 definitions. If SIRS was due to a documented infection, patients were classified as sepsis patients. Severe sepsis was defined as sepsis accompanied by organ dysfunction, i. e., defined as recommended [26] for the 1992 sepsis definitions, or greater than two points in one organ system using the SOFA score. Septic shock was defined as sepsis together with hypotension despite adequate fluid resuscitation. Sepsis was defined due to the 2003 definitions [15], if at least two out of the enlarged list of general and inflammatory variables were fulfilled, i.e., temperature, heart rate, respiratory rate, significant edema, positive fluid balance, hyperglycemia (plasma glucose > 120 mg/dl), white blood cell count (WBC), and plasma C-reactive protein. Severe sepsis was defined as sepsis plus organ dysfunction according to the limitations for organ dysfunction variables and tissue perfusion variables (hyperlactatemia) given in the 2003 sepsis definitions publication [15]. The reason for the two sepsis definitions is that the frequency and mortality rates of various sepsis severity stages differ if defined by the 1992 or the 2003 definitions within the same patient population [27]. The 1992 definitions may under-classify patients with severe sepsis. Thus, transferring recommendations such as those of the SSC guidelines drawn from data sets regarding severity of sepsis generated with the 1992 definitions to the same population applying the 2003 definitions may be misleading. Therefore, in the present study, sepsis was defined using the original 1992 ACCP/SCCM [24] and the revised 2003 SCCM/ESICM/ACCP/ATS/SIS sepsis definitions [15]. In the 2003 definitions, hyperglycemia is defined as plasma glucose > 120 mg/dl in the absence of diabetes and is one of the general variables in the diagnostic criteria for sepsis. Therefore, we expected different subgroups of patients in our patient collective when defining sepsis with the 1992 or the 2003 definitions. Acute kidney injury was defined as urine output < 0.5 ml/kg for more than 2 hours [15], and/or increase in creatinine > 44 umol/l [15] and/or extracorporeal renal support [15]. Hydrocortisone was used in adult septic shock according to the SSC guidelines [1,2], i. e., after blood pressure was identified to be poorly responsive to fluid resuscitation and vasopressor therapy.

The following patients were excluded in the final evaluation: patients with unfavorable prognosis, patients < 18 years (because SAPS II score [23] and the ACCP/SCCM sepsis definitions [24] have been developed for patients ≥ 18 years, and the SOFA score [26] for patients ≥ 12 years), patients with insulin dependent diabetes mellitus, patients with previous diagnosis of diabetes mellitus, and patients with a stay < 72 hours on the ICU in analogy to previous studies [4].

Routine blood glucose measurements were performed by a blood gas analyzer (Radiometer Copenhagen GmbH, Willich, Germany) according to the guidelines of the "Surviving Sepsis Campaign" [1,2]. Thus, in our department, glucose measurements are performed routinely every 1 - 2 hrs (4 hrs when stable) in patients receiving intravenous insulin, and every 30 to 60 minutes in patients with glucose values < 80 mg/dl. In severely ill patients without insulin administration, blood glucose was measured at least four times a day.

### Statistical analysis

Data comprised clustered observations because of repeated measures taken during each patient's course in the ICU. Therefore, analyses of associations between blood glucose concentrations < 80 mg/dl/blood glucose concentrations ≥ 150 mg/dl and demographic/clinical variables were performed in a logistic regression adjusted for clustered observations.

In a first step, separately for each of the two outcome variables (blood glucose concentrations < 80 mg/dl vs 80 - < 150 mg/dl, and blood glucose concentrations ≥ 150 mg/dl vs 80 - < 150 mg/dl) and any of the explanatory variables, associations were assessed by logistic regression. Crude odd ratios (ORs) with 95% confidence interval (CI) and p-values were calculated.

In a second step, for each outcome variable, the explanatory variables were assessed simultaneously, and relevant factors were identified by backward elimination in a multiple logistic regression.

## Results

During the period dating from 01/2007 to 12/2008, 1637 postoperative/posttraumatic critically ill patients were admitted to the ICU. A total of 804 patients met the inclusion criteria with an ICU stay > 72 hours and were eligible for final evaluation.

Baseline characteristics of these patients were as follows. The median of age was 65 (range 18 - 98, mean 61.0, SD 17.1). Out of these 804 patients, 241 (30%) were female, 84 (10.5%) were non-survivors, 225 (28%) were admitted due to neurosurgery, 217 (27%) due to abdominal surgery, 193 (24%) due to vascular surgery, and 169 (21%) due to trauma surgery. The median SAPS II score on admission was 32 (range 6 - 82, mean 34.5, SD 15.9).

Characteristics during the course on the ICU were as follows. Insulin was applied in 642 (79.9%) of the 804 patients, steroids in 265 (33.0%), adrenaline in 191 (23.8%), noradrenaline in 564 (70.2%). Extracorporeal renal support was performed in 84 (10.5%) patients. 478 (60.0%) patients had severe SIRS/sepsis due to the 1992 definitions, and 705 (88.7%) due to the 2003 definitions. The maximal MELD score was 7 in median (range -46 - 69, mean 9.6, SD 9.98). The maximal SOFA score was 6 in median (range 0 - 18, mean 6.5, SD 3.3).

Complete information regarding the daily minimal and maximal blood glucose concentrations, insulin administration, demographic/clinical variables and scores were available in 7409 observations in total, of these 2273 without and 5136 with insulin administration. The number of observations within distinct blood glucose ranges in all patients are presented in Table 1. Blood glucose concentrations < 80 mg/dl occured in 10.9% of our observations.

**Table 1:** Distribution of the number of observations within various blood glucose ranges

	Observations (all, n = 7,409)	
**Blood glucose (mg/dl)**		**% of all**

< 40	65	0.9%
40 to <60	122	1.6%
60 to <80	623	8.4%
80 to <150	3583	48.4%
≥ 150	3016	40.7%

To convert the values for glucose to millimoles per litre, multiply by 0.05551.

To identify variables strongly associated with blood glucose concentrations < 80 mg/dl or ≥ 150 mg/dl, crude odds ratios (OR) with 95% confidence intervals (95% CI) and p-values were calculated and are presented in Table 2. The variables associated with an increased risk of blood glucose concentrations < 80 mg/dl were: age, female sex, SAPS II > 36, MELD > 9, SOFA > 4, application of adrenaline, noradrenaline, steroids or insulin. Those variables associated with an increased risk of blood glucose concentrations ≥ 150 mg/dl were: age, neurosurgery compared to all other types (abdominal, vascular, trauma surgery), SAPS II > 36, sepsis defined by the 1992 or the 2003 definitions, MELD > 9, SOFA > 4, and administration of adrenaline, noradrenaline, steroids or insulin.

**Table 2:** Crude OR (95% CI) of factors associated with blood glucose concentrations < 80 mg/dl or ≥ 150 mg/dl

	BG < 80 vs 80 ≤ BG < 150		BG ≥150 vs 80 ≤ BG <150	
	**OR (95% CI)**	**P value**	**OR (95% CI)**	**P value**

Age (per year)	**1.02 (1.01; 1.03)**	**<0.001**	**1.02 (1.01; 1.02)**	**<0.001**
Gender (female vs male)	**1.4 (1.1; 1.8)**	**0.014**	1.1 (0.95; 1.4)	0.148
Neurosurgery	1.0	0.324	**1.0**	**<0.001**
Abdominal surgery	0.8 (0.6; 1.1)		**0.7 (0.6; 0.9)**	
Vascular surgery	0.8 (0.6; 1.1)		**0.7 (0.6; 0.9)**	
Trauma surgery	0.7 (0.5; 1.03)		**0.5 (0.4; 0.7)**	
SAPS II (>36 vs ≤36)	**1.5 (1.2; 1.8)**	**<0.001**	**1.5 (1.3; 1.7)**	**<0.001**
Sepsis 1992 severe/shock (yes vs no)	1.2 (0.99; 1.4)	0.068	**1.4 (1.2; 1.6)**	**<0.001**
Sepsis 2003 severe/shock (yes vs no)	1.1 (0.9; 1.3)	0.355	**1.2 (1.04; 1.3)**	**0.012**
Extracorporeal renal replacement therapy (yes vs no)	1.3 (0.9; 1.8)	0.124	1.3 (0.95; 1.7)	0.103
MELD (>9 vs ≤9)	**1.3 (1.1; 1.7)**	**0.008**	**1.3 (1.1; 1.5)**	**0.005**
SOFA (>4 vs ≤4)	**1.4 (1.2; 1.7)**	**<0.001**	**1.5 (1.3; 1.7)**	**<0.001**
Adrenaline (yes vs no)	**1.7 (1.9; 2.4)**	**0.004**	**1.3 (1.02; 1.6)**	**0.031**
Noradrenaline (yes vs no)	**1.7 (1.4; 2.0)**	**<0.001**	**1.7 (1.5; 2.0)**	**<0.001**
Steroids (yes vs no)	**1.8 (1.4; 2.2)**	**<0.001**	**2.0 (1.7: 2.3)**	**<0.001**
Insulin (yes vs no)	**2.4 (1.9; 3.0)**	**<0.001**	**2.8 (2.4; 3.2)**	**<0.001**

BG = blood glucose; CI = confidence interval; IIT = intensive insulin therapy; MELD = model of end stage liver disease; OR = odds ratio; SAPS II = Simplified Acute Physiology Score; SOFA = Sequential Organ Failure Assessment.

Number of data sets: see Table 1. ORs with p < 0.05 are highlighted in bold numbers.

To convert the values for glucose to millimoles per litre, multiply by 0.05551.

For comparison with the other types of surgery, the risk has been set at 1 for neurosurgery.

Multiple logistic regression with backward elimination was performed to simultaneously assess and identify variables strongly associated with blood glucose concentrations < 80 mg/dl or ≥ 150 mg/dl. Thereby, increasing age, application of noradrenaline, steroids or insulin were strongly associated with blood glucose concentrations < 80 mg/dl (Table 3). In addition to these variables, neurosurgery compared to all other types of surgery, and sepsis as defined by the 1992 definitions were strongly associated with blood glucose concentrations ≥ 150 mg/dl (Table 3).

**Table 3:** Multiple logistic regression with backward elimination

	BG < 80 vs 80 ≤ BG < 150		BG ≥ 150 vs 80 ≤ BG < 150	
	**OR (95% CI)**	**P value**	**OR (95% CI)**	**P value**

Age (per year)	1.02 (1.01; 1.03)	<0.001	1.01 (1.01; 1.02)	<0.001
Gender (female vs male)	--		--	
Neurosurgery	--		1.0	<0.001
Abdominal surgery			0.6 (0.5; 0.7)	
Vascular surgery			0.7 (0.6; 0.9)	
Trauma surgery			0.6 (0.5; 0.8)	
SAPS II (>36 vs ≤36)	--		--	
Sepsis 1992 severe/shock (yes vs no)	--		1.2 (1.1; 1.4)	0.003
Sepsis 2003 severe/shock (yes vs no)	--		--	
Extracorporeal renal replacement therapy (yes vs no)	--		--	
MELD (>9 vs ≤9)	--		--	
SOFA (>4 vs ≤4)	--		--	
Adrenaline (yes vs no)	--		--	
Noradrenaline (yes vs no)	1.4 (1.2; 1.8)	<0.001	1.4 (1.2; 1.6)	<0.001
Steroids (yes vs no)	1.3 (1.003; 1.7)	0.047	1.4 (1.2; 1.7)	<0.001
Insulin	2.1 (1.7; 2.6)	<0.001	2.4 (2.0; 2.7)	<0.001

OR (95% CI) of factors associated with blood glucose concentrations < 80 mg/dl or ≥ 150 mg/dl.

BG = blood glucose; CI = confidence interval; IIT = intensive insulin therapy; MELD = model of end stage liver disease; OR = odds ratio; SAPS II = Simplified Acute Physiology Score; SOFA = Sequential Organ Failure Assessment.

Number of data sets: see Table 1.

To convert the values for glucose to millimoles per litre, multiply by 0.05551.

For comparison with the other types of surgery, the risk has been set at 1 for neurosurgery.

The relationship of clinical outcome and blood glucose concentrations < 80 mg/dl or ≥ 150 mg/dl is given in Table 4. Compared to patients with blood glucose levels always within the target range of 80 to 150 mg/dl, patients with episodes of blood glucose concentrations < 80 mg/dl, ≥ 150 mg/dl, as well as < 80 mg/dl and ≥ 150 mg/dl, in addition, revealed an increased risk of death (p = 0.009).

**Table 4:** Blood glucose variability and outcome

80 ≤ BG < 150 (mg/dl)	Total patients	Nonsurvivors (%)	OR (95% CI)	P value
always	118	4 (3.4%)	1.0	0.009
plus BG < 80	26	2 (7.7%)	2.4 (0.4; 13.7)	
plus BG ≥ 150	450	45 (10.0%)	3.2 (1.1; 9.0)	
plus BG < 80 and BG ≥ 150	210	33 (15.7%)	5.3 (1.8; 15.4)	
	804	84 (10.5%)		

BG = blood glucose; CI = confidence interval.

To convert the values for glucose to millimoles per litre, multiply by 0.05551.

## Discussion

The present study reveals that the most relevant factors associated with blood glucose concentrations < 80 mg/dl or ≥ 150 mg/dl are age and application of noradrenaline, steroids or insulin. In addition, neurosurgery and severe sepsis/shock are variables strongly associated with blood glucose concentrations ≥ 150 mg/dl.

Patients who underwent neurosurgery and those in septic shock [1,2], are frequently treated with noradrenaline and steroids. In separate analyses of explanatory variables (Table 2), severe sepsis/septic shock was associated with increased risk of blood glucose concentrations ≥ 150 mg/dl. Hyperglycemia, i. e. plasma glucose > 120 mg/dl in the absence of diabetes, is one of the broadened diagnostic criteria for sepsis in the 2003 definition. The frequencies of severe sepsis and septic shock were higher and mortality rates lower when the 2003 definitions (lower thresholds for the definition of organ dysfunctions [15]) were applied instead of the 1992 definitions although it was in the same population of critically ill surgical patients [27]. Elderly neurosurgical patients requiring noradrenaline to increase intracranial perfusion pressure and steroids to reduce edema and swelling, as well as elderly patients with more severe organ dysfunctions and in septic shock who were treated with noradrenaline and steroids according to the "Surviving Sepsis Campaign" guidelines [1,2] are at higher risk for blood glucose concentrations ≥ 150 mg/dl.

In a recent meta-analysis, there was no survival benefit regarding tight glucose control with intensive insulin therapy (IIT) in all analyzed 13,567 patients (OR 0.93, 95% CI 0.83 - 1.04), however, in the subset of surgical patients (OR 0.63, 95% CI 0.44 - 0.90) [8] there was a significant survival benefit. In the perioperative concept of IIT, the greatest reduction in mortality in surgical ICU patients with sepsis and receiving mechanical ventilation involved deaths due to multiple-organ failure [3]. In these patients with severe sepsis it was speculated that the higher rate of severe hypoglycemia in the IIT group [3] was due to renal or liver dysfunction [6]. In the present study, surgical patients with renal dysfunction assessed as extracorporeal renal replacement therapy were not associated with an increased risk of blood glucose concentrations < 80 mg/dl or ≥ 150 mg/dl (Table 2, 3). This is in contrast to medical-surgical ICU patients, where hemodialysis was a predictor of hypoglygemia [10] and renal insufficiency was an independent risk factor for severe hypoglycemia [14]. In the present study, severity of liver dysfunction was assessed by the MELD score and of organ dysfunctions by the SOFA score. In separate analyses (Table 2), a MELD score > 9 and a SOFA score > 4 were associated with a higher risk of blood glucose concentrations < 80 mg/dl or ≥ 150 mg/dl, however, this association was not found by multiple logistic regression (Table 3). In medical-surgical ICU patients, female gender was one of the main predictors of hypoglycemia [10]. The higher risk of blood glucose concentrations < 80 mg/dl with age (Tables 2, 3) and female gender (Table 2) in the present study are most probably due to an impaired counterregulatory response [12]. The higher risk of hypoglycemia may be due to the lower counterregulatory hormone threshold for hypoglycemia in women than in men, i. e., especially adrenaline, noradrenaline and growth hormone response [12]. The association of blood glucose concentrations < 80 mg/dl with administration of noradrenaline and steroids was surprising. In a rat model of continuous endotoxin infusion resembling some of the metabolic and cardiovascular constellations of human sepsis, a decreased number of hepatic plasma membrane alpha 1-adrenergic receptors were found [28]. This might in part explain why a comparable subset of our patients might not have developed blood glucose concentrations ≥ 150 mg/dl but < 80 mg/dl in association with continuous noradrenaline infusion. On the other hand, hyperglycemia due to insulin resistance has been reported in human sepsis. Adipokine levels were extensively altered in patients with severe sepsis and shock [29]. Adiponectin, the prototype of an anti-inflammatory and insulin-sensitizing adipocytokine, was diminished in these patients [29]. Out of the insulin-resistance mediating factors, plasminogen activator inhibitor-1 (PAI-1), MCP-1, IL-6, IL-8, IL-10 and tumour necrosis factor (TNF)-α were significantly elevated in these patients. All significant changes were shifted in the same direction as in obese subjects and patients with type 2 diabetes. These results may help to explain insulin resistance in critically ill patients and patients with systemic inflammatory response syndrome. After diagnosis of severe sepsis and shock, serum adiponectin levels and hydrocortisone correlated positively with insulin demand, and noradrenaline demand negatively with male adiponectin levels [30]. Septic shock causes a massive dilation of the peripheral vascular system, promoted by inflammatory cytokines and microbial toxins. Thus, the dosages of noradrenaline needed to provide a sufficient mean arterial pressure can be used to assess the degree of deterioration of the circulation (Sequential Organ Failure Assessment, SOFA score) for severity of organ dysfunction [26]. Thus, an increase in noradrenaline demand may reflect a clinical situation with changes in adipokines resulting in insulin resistance. Taken together, these data in septic patients may explain why we observed a positive association of noradrenaline with blood glucose concentrations ≥ 150 mg/dl. On the other hand, adipokine panels resulting in overwhelming insulin sensitivity might set patients at risk of blood glucose concentrations < 80 mg/dl. In this constellation, we might get an association of steroid and noradrenaline treatment with blood glucose concentrations < 80 mg/dl, which is not due to the effects of steroids or noradrenaline per se. The variables SAPS II > 36, MELD > 9, and SOFA > 4 in the present study were associated with an increased risk of blood glucose concentrations < 80 mg/dl reflecting patients with greater severity of disease and organ dysfunctions. One third of our patients were neurosurgical patients. Neurosurgical patients are often treated with noradrenaline to increase intracranial perfusion pressure and steroids to reduce edema and swelling. Thus, the pathopysiological reasons and the underlying adipokine pattern during steroid or noradrenaline treatment in our heterogenous patient groups may differ profoundly. This might explain why we found noradrenaline and steroid treatment as risk factors for both, blood glucose concentrations ≥ 150 mg/dl and < 80 mg/dl.

The blunted ability of neutrophils to adapt to physiological hyperinsulinemia in older people (69 +/- 4 years) may compromise the anti-infective response [31], and may contribute to sepsis. Taken together, elderly female patients with extracorporeal renal replacement therapy and/or liver dysfunction, especially under IIT, may be at increased risk of hypoglycemia.

The "Surviving Sepsis Campaign" guidelines for the management of severe sepsis and septic shock recommended intensive insulin therapy (IIT) targeting of glucose levels to the < 150 mg/dl range [1,2]. In the present study, 48.4% of minimal and maximal blood glucose data sets per day were in the target range of 80 to < 150 mg/dl (Table 1). This is comparable with a previous study in a mixed medical-surgical ICU with strict glycemic control, in which less than 50% of patients were within the target range with a considerable overlap between the intensive and the standard insulin group [10]. In the present study, severity of disease was assessed by SAPS II score. In separate analysis only (Table 2), SAPS II was associated with increased risk for blood glucose concentrations < 80 mg/dl or ≥ 150 mg/dl, however, not in multiple logistic regression (Table 3). Severity of illness reflected by the APACHE II score was one of the factors associated with the development of severe hypoglycemia in medical, surgical, and cardiac ICU patients in multiple logistic regression [10,14]. Thus, the severity of disease may put patients at risk of not falling within the recommended target range of 80 to 150 mg/dl blood glucose concentration.

In the present study, the overall hypoglycemia rates observed with blood glucose concentrations < 40 mg/dl in 0.9% and < 80 mg/dl in 10.9% (Table 1) were low, furthermore the administration of insulin was associated with an increased risk of blood glucose concentrations < 80 mg/dl (OR 2.1; 95% CI 1.7 - 2.6) (Table 3). A comparison with hypoglycemia rates in the literature is difficult since hypoglycemia rates with IIT might be related to differences in patient populations, severity of illness, protocols, blood glucose targets, definitions of hypoglycemia, proportion of diabetic patients, ICU length of stay or duration of therapy. For example, the rates of hypoglycemia (≤ 40 mg/dl) were 0.5% [5], 1.7% [32] and 2.7% [33] in the control group, and 6.8% [5], 8.5% [32], 8.7% [33] and 16% [10] in the IIT group, respectively, in mixed surgical/medical ICUs. Taken together, in a recent meta-analysis, IIT was associated with a high risk (OR 6.0, 95% CI 4.5 - 8.5) for hypoglycemia < 40 mg/dl [8].

A comparison to patients with blood glucose levels always within the target range of 80 to 150 mg/dl, patients with episodes of blood glucose concentrations < 80 mg/dl, of ≥ 150 mg/dl, or of both, revealed an increased risk of death (Table 4). It has been suggested that quadrupling the rate of severe hypoglycemia and doubling the mortality attributable to severe hypoglycemia would erase the survival benefit of IIT in medical, surgical, and cardiac patients [14]. It remains unclear, whether hypoglycemia is associated with worse outcome or is simply a marker of severity of illness [10]. Spontaneous hypoglycemia without insulin therapy might be even more dangerous, resulting in a 1.7-fold increase in mortality [34], because it is often detected late, furthermore, the duration of the episodes are longer due to less frequent monitoring than with IIT.

Thus, it is of high clinical relevance to know that beyond insulin therapy, the most relevant factors associated with hypoglycemia were noradrenaline infusion, steroids and age (Table 3). To prevent deleterious hypoglycemia, a higher target range of 140 - 180 instead of < 150 mg/dl in critically ill patients was recently suggested [35,36].

The strengths of our study are that our ICU has a high nursing staff to patient ratio and is operated 24/7 by an on-site Critical Care Board-certified intensivist, and that intervention-related risk factors for blood glucose concentrations ≥ 150 mg/dl were evaluated. On the other hand, our study has several limitations. It is a single center trial. Other factors include adherence to the intensive insulin therapy protocol, the frequency of glucose measurements, the amount of average calories/day, the insulin dose, and the small number of hypoglycemic episodes < 40 mg/dl, however most of the aforementioned limitations apply to many of the recently published studies concerning IIT as well.

## Conclusions

The present study shows that the most relevant factors for blood glucose concentrations < 80 mg/dl in critically ill surgical patients were age, administration of noradrenaline, steroids and insulin. The most relevant factors for blood glucose concentrations ≥ 150 mg/dl were age, neurosurgery, severe sepsis/shock as defined by the 1992 definitions, steroids, noradrenaline and insulin. Thus, physicians and nurses have to be alert to these factors to avoid blood glucose concentrations < 80 mg/dl or hypoglycemia induced harm, especially in subgroups of patients with a combination of these most relevant factors.

## Competing interests

The authors declare that they have no competing interests.

## Authors' contributions

MW, MK, BH, PR and MG participated in study conception, study design, data analysis, interpretation and drafting of the manuscript.

MT and MW participated in programming the computer-assisted scoring systems and data base, data acquisition, data analysis and interpretation of the manuscript.

All authors read and approved the final manuscript.
